# Supplementary material for: In vitro reconstitution of chromatin domains shows a role for nucleosome positioning in 3D genome organization
Source: Nat Genet. 2024 Jan 30;56(3):483–92. doi: 10.1038/s41588-023-01649-8 (PMC10937381; doi:10.1038/s41588-023-01649-8)
Supplement: Supplementary file 1 — Supplementary Text. [file 41588_2023_1649_MOESM1_ESM.pdf]

nature genetics

Article

https://doi.org/10.1038/s41588-023-01649-8

In vitro reconstitution of chromatin domains shows a role for nucleosome positioning in 3D genome organization

In the format provided by the authors and unedited

Supplementary Text

Expression and purification of yeast histone octamers

Yeast histone co-expression plasmids[62] were expressed in *Escherichia coli* BL21(DE3) RIL cells (Supplementary Table 1). Cells were grown until OD_{600} reached 0.6 in 2 x 2 L LB medium with 100 μg/mL spectinomycin, 100 μg/mL ampicillin and 20 μg/mL chloramphenicol. Expression of histones was induced by addition of 0.8 mM isopropyl 1-thio-β-d-galactopyranoside (IPTG) for 3 hours at 37ºC. Cells were harvested, resuspended in 60 mL lysis buffer (20 mM Tris-Cl pH 7.6, 500 mM NaCl, 0.1 mM EDTA and 1x Protease Inhibitor Cocktail containing 0.284 μg/mL leupeptin, 1.37 μg/mL pepstatin A, 0.17 mg/mL PMSF, 0.33 mg/mL benzamidine), flash frozen and stored at -80ºC.

Thawed cells were lysed by sonication on ice with a Branson Sonifier (4 x 4 minutes, 5 seconds on pulses, 5 seconds off pulses at 50% duty cycle). The lysate was cleared for 45 minutes at 89,000 g at 4ºC (Sorvall LYNX 6000 centrifuge) and the supernatant was filtered with a 0.45 μM syringe filter. The cleared supernatant was loaded onto an equilibrated HiTrap Heparin 5 mL HP column (Cytiva). The column was washed with buffer A (20 mM Tris-Cl pH 7.6, 500 mM NaCl, 0.1 mM EDTA) followed by gradient elution over 14 CV of buffer A to buffer B (20 mM Tris-Cl pH 7.6, 2 M NaCl). Peak fractions were pooled and further purified by size exclusion chromatography with a Superdex 200 10/300 Increase column (Cytiva) equilibrated with 20 mM HEPES pH 7.5, 2 M NaCl. Peak fractions were pooled, concentrated and stored at -20°C in 50% glycerol.

Expression and purification of ATP-dependent chromatin remodeling enzymes

Chd1 and ISW2

Hi5 cells (1.2 L; Supplementary Table 1) were grown in ESF-921 media (Expression Systems) and infected with V1 virus for full-length Chd1 (tagged with a N-terminal 6 × His tag, followed by a MBP tag, and a tobacco etch virus protease cleavage site) or ISW2 (Isw2 tagged with an N-terminal 6 × His tag, followed by an MBP tag, and a tobacco etch virus protease cleavage site) for protein expression. Cells were grown for 72 hours at 27 °C and subsequently harvested by centrifugation (238 g, 4 °C, 30 minutes). Cell pellets were resuspended in lysis buffer (20 mM Na·HEPES pH 7.4, 300 mM NaCl, 10% (v/v) glycerol, 1 mM DTT, 30 mM imidazole pH 8.0, 0.284 µg/mL leupeptin, 1.37 µg/mL pepstatin A, 0.17 mg/mL PMSF, 0.33 mg/mL benzamidine), flash frozen and stored at -80 °C.

Chd1 was purified as previously described[14]. For ISW2 purification, frozen cell pellets were thawed, lysed by sonication and cleared by centrifugation (18,000 g, 4 °C, 30 minutes and 235,000 g, 4 °C, 60 minutes). The supernatant was filtered with 0.8-µm syringe filters

(Millipore) and applied onto a HisTrap HP 5 mL column (Cytiva). The column was washed first with lysis buffer, then with high salt buffer (20 mM Na·HEPES pH 7.4, 1 M NaCl, 10% (v/v) glycerol, 1 mM DTT, 30 mM imidazole pH 8.0, 0.284 µg/mL leupeptin, 1.37 µg/mL pepstatin A, 0.17 mg/mL PMSF, 0.33 mg/mL benzamidine), and finally with lysis buffer. Protein was eluted with 20 mM Na·HEPES pH 7.4, 300 mM NaCl, 10% (v/v) glycerol, 1 mM DTT, 500 mM imidazole pH 8.0 onto a prepacked Amylose column (NEB). HisTrap HP column was disconnected, the Amylose column was washed with 20 mM Na·HEPES pH 7.4, 150 mM NaCl, 10% (v/v) glycerol, 1 mM DTT and protein was eluted with wash buffer containing 4% Maltose. Protein containing fractions were pooled and subjected to TEV protease digestion for 6 h. 10 mM Imidazole was added and the sample was applied to a HisTrap HP 5 mL which was attached to a HiTrap Q HP 5 mL column. HisTrap was detached, Q column was washed and eluted with a gradient to 100% high salt buffer (20 mM Na·HEPES pH 7.4, 1 M NaCl, 10% (v/v) glycerol, 1 mM DTT). Sample was concentrated in a 50 kDa concentrator (Amicon, Merck) and loaded onto a Sepharose6 10/300 Increase pre-equilibrated in 20 mM Na·HEPES pH 7.4, 300 mM NaCl, 10% (v/v) glycerol, 1 mM DTT. Protein containing fractions were pooled, concentrated, flash frozen and stored at -80°C.

INO80

INO80 was purified endogenously from an overexpression strain (Supplementary Table 1)[64]. 6 × 2 L of prewarmed YP medium with 2% Raffinose was inoculated at OD_{600} 0.1, grown for ~20 hours at 30°C at 150 rpm and induced around OD_{600} 5 with 2% Galactose (final concentration). After 3 hours induction, cells were harvested, resuspended in 100 mL of lysis buffer (50 mM HEPES-KOH pH 7.5, 500 mL KCl, 1 mM EDTA, 8 mM MgCl2, 0.1% NP-40, 20% Glycerol, 4 mM DTT, 0.496 µg/mL leupeptin, 2.74 µg/mL pepstatin A, 0.34 mg/mL PMSF, 0.66 mg/mL benzamidine) and frozen dropwise into liquid nitrogen. Popcorn was subjected to cryo-milling (Spex Freezer/Mill 6875D) and stored at -80°C.

All following steps were performed at 4°C unless stated otherwise. Powder was thawed in a 30°C-waterbath, then centrifuged at 30,000 g for 15 minutes and brought to a final concentration of 400 mM KCl. Lysate was subjected again to centrifugation at 90,000 g for 45 minutes. Cleared supernatant was incubated with 600 μL prewashed FLAG-M2 beads for 1 h. Beads were washed with wash buffer (25 mM HEPES- KOH pH 8, 200 mM KCl, 10% Glycerol, 0.001% IGEPAL-CA630, 2 mM $MgCl_2$, 1 mM DTT) and eluted with wash buffer + 0.22 mg/mL FLAG peptides. Elution was loaded onto a HiTrap Q HP 1 mL column equilibrated with 25 mM HEPES- KOH pH 8, 200 mM KCl, 10% Glycerol, 1 mM $MgCl_2$, 1 mM DTT. The column was

washed and then eluted with a gradient to 1 M KCl. INO80 containing fractions were pooled, concentrated, flash frozen and stored at -80°C.

RSC

RSC was purified from yeast using the TAP-tag method. Rsc2-TAP-tagged yeast strain (Supplementary Table 1) was fermented in 250 L 3% YEP broth (w/v, Formedium) supplemented with 2% glucose, 50 g L^{-1} ampicillin sodium salt and 12.5 g L^{-1} tetracycline hydrochloride until OD_{600} 10. Cells were harvested, washed with ice-cold water and resuspended 1:1 (v/v) in Lysis Buffer (50 mM K·HEPES pH 7.6 at 4°C, 700 mM KOAc, 1 mM MgCl2, 5% Glycerol, 1 mM DTT, 2x PI (0.56 μg/mL leupeptin, 2.74 μg/mL pepstatin A, 0.34 mg/mL phenylmethylsulfonyl fluoride, 0.66 mg/mL benzamidine)). Resuspended cells were frozen dropwise into liquid nitrogen, subjected to cryo-milling (Spex Freezer/Mill 6875D) and stored at -80°C.

All following steps were performed at 4°C unless stated otherwise. Powder was thawed in a 30°C-waterbath and slurry was first centrifuged at 14,000 g for 15 minutes followed by ultracentrifugation at 200,000 g for 105 minutes. Clear phase was taken and supplemented with 50 mM KOAc. Then, the lysate was incubated with prewashed IgG Sepharose 6 Fast Flow resin (Cytiva) for 3 h. Resin was collected by centrifugation and washed 2x with Wash buffer (40 mM K·HEPES pH 7.6, 250 mM KAc, 10% Glycerol, 0.5 mM DTT, 10 mM EDTA pH 8, 1x PI) and 1x with TEV-Elution Buffer (40 mM K·HEPES pH 7.6, 200 mM KAc, 10% Glycerol, 0.5 mM DTT, 10 mM EDTA pH 8). Protein was eluted by addition of TEV-Elution buffer containing 10 μg/mL TEV protease and incubation for 2 hours at 15°C with gentle agitation. Elution was loaded onto a pre-equilibrated 1 mL HiTrap Q HP column (Cytiva), washed and eluted over 40 CV from Buffer A to B (Buffer A: 40 mM HEPES-KOH pH 7.6, 200 mM KAc, 10% Glycerol, 1 mM DTT, Buffer B: 40 mM K·HEPES, 1.5 M KAc, 10% Glycerol, 1 mM DTT). RSC-containing fractions were pooled, concentrated, flash frozen and stored at -80°C.
