# Supplementary material for: In vitro reconstitution of chromatin domains shows a role for nucleosome positioning in 3D genome organization
Source: Nat Genet. 2024 Jan 30;56(3):483–92. doi: 10.1038/s41588-023-01649-8 (PMC10937381; doi:10.1038/s41588-023-01649-8)
Supplement: Supplementary file 2 — Reporting Summary [file 41588_2023_1649_MOESM2_ESM.pdf]

nature portfolio

Corresponding author(s): Elisa Oberbeckmann, A. Marieke Oudelaar

Last updated by author(s): Dec 2, 2023

Reporting Summary

Nature Portfolio wishes to improve the reproducibility of the work that we publish. This form provides structure for consistency and transparency in reporting. For further information on Nature Portfolio policies, see our Editorial Policies and the Editorial Policy Checklist.

Statistics

For all statistical analyses, confirm that the following items are present in the figure legend, table legend, main text, or Methods section.

n/a	Confirmed	
☐	☒	The exact sample size (n) for each experimental group/condition, given as a discrete number and unit of measurement
☐	☒	A statement on whether measurements were taken from distinct samples or whether the same sample was measured repeatedly
☐	☒	The statistical test(s) used AND whether they are one- or two-sided<br>*Only common tests should be described solely by name; describe more complex techniques in the Methods section.*
☒	☐	A description of all covariates tested
☒	☐	A description of any assumptions or corrections, such as tests of normality and adjustment for multiple comparisons
☐	☒	A full description of the statistical parameters including central tendency (e.g. means) or other basic estimates (e.g. regression coefficient) AND variation (e.g. standard deviation) or associated estimates of uncertainty (e.g. confidence intervals)
☐	☒	For null hypothesis testing, the test statistic (e.g. F, t, r) with confidence intervals, effect sizes, degrees of freedom and P value noted<br>*Give P values as exact values whenever suitable.*
☒	☐	For Bayesian analysis, information on the choice of priors and Markov chain Monte Carlo settings
☒	☐	For hierarchical and complex designs, identification of the appropriate level for tests and full reporting of outcomes
☐	☒	Estimates of effect sizes (e.g. Cohen's d, Pearson's r), indicating how they were calculated

Our web collection on statistics for biologists contains articles on many of the points above.

Software and code

Policy information about availability of computer code

Data collection	Illumina NextSeq 550.
Data analysis	Trim Galore v.0.3.1; FLASH v.1.2.11; BLAT v.35; Bowtie v.1.2.1.1; Bowtie2 v.2.3.5; GenomicAlignments v.1.30.0; igv v.2.8.6; MEME 5.5.2; MACS2 2.2.8; HiC-Pro v.2.11.1; cooler v.0.8.11; cooltools v.0.5.1; hiCExplorer v.3.6; MCC pipeline v.1 (https://github.com/jojdavies/Micro-Capture-C); R Studio (v.2023.030+386; R v.4.1.3); see Methods section for full details.

For manuscripts utilizing custom algorithms or software that are central to the research but not yet described in published literature, software must be made available to editors and reviewers. We strongly encourage code deposition in a community repository (e.g. GitHub). See the Nature Portfolio guidelines for submitting code & software for further information.

Data

Policy information about availability of data

All manuscripts must include a data availability statement. This statement should provide the following information, where applicable:

- Accession codes, unique identifiers, or web links for publicly available datasets
- A description of any restrictions on data availability
- For clinical datasets or third party data, please ensure that the statement adheres to our policy

All raw sequencing data and processed data generated in this study are available for download at http://www.ncbi.nlm.nih.gov/geo/ via GEO accession number

GSE220647. In vivo Micro-C data are available via GEO accession numbers GSM2262329, GSM2262330, and GSM2262331, and in vivo ChIP-seq data for Abf1 and Reb1 via GEO accession numbers GSM4449154, GSM4449823, GSM2916412, and GSM2916410.

Human research participants

Policy information about studies involving human research participants and Sex and Gender in Research.

Reporting on sex and gender	n/a
Population characteristics	n/a
Recruitment	n/a
Ethics oversight	n/a

Note that full information on the approval of the study protocol must also be provided in the manuscript.

Field-specific reporting

Please select the one below that is the best fit for your research. If you are not sure, read the appropriate sections before making your selection.

☒ Life sciences ☐ Behavioural & social sciences ☐ Ecological, evolutionary & environmental sciences

For a reference copy of the document with all sections, see nature.com/documents/nr-reporting-summary-flat.pdf

Life sciences study design

All studies must disclose on these points even when the disclosure is negative.

Sample size	No statistical methods were used to pre-determine sample sizes but our sample sizes are similar to those reported in previous publications. The data presented in the manuscript represent the averages of multiple replicates as stated in each of the figures and described in detail in the Methods section. These sample sizes were chosen to generate data at sufficient depth and assess differences between conditions robustly. These sample sizes are sufficient, since the observed effects of interest are clearly detectable between conditions and robust across replicates.
Data exclusions	No data were excluded.
Replication	Experiments were performed independently at least two times as described in detail in the Methods section and all attempts were successful.
Randomization	Since the researchers need to know the experimental condition in order to perform the experiments successfully, randomization is not relevant for our study.
Blinding	All samples were analyzed with the same pipeline, in which interactions are detected by scripts without interference of the researchers. Since potential expectations of the researchers cannot influence the data analysis and results, blinding is not relevant to this study.

Reporting for specific materials, systems and methods

We require information from authors about some types of materials, experimental systems and methods used in many studies. Here, indicate whether each material, system or method listed is relevant to your study. If you are not sure if a list item applies to your research, read the appropriate section before selecting a response.

Materials & experimental systems

n/a	Involved in the study	
☐	☒	Antibodies
☐	☒	Eukaryotic cell lines
☒	☐	Palaeontology and archaeology
☒	☐	Animals and other organisms
☒	☐	Clinical data
☒	☐	Dual use research of concern

Methods

n/a	Involved in the study	
☐	☒	ChIP-seq
☒	☐	Flow cytometry
☒	☐	MRI-based neuroimaging

Antibodies

Antibodies used	Anti-FLAG M2 Affinity Gel (A2220, Merck) was used for protein purification.
Validation	Anti-FLAG M2 Affinity Gel (A2220, Merck) was used for protein purification and thus required no additional validation.

Eukaryotic cell lines

Policy information about cell lines and Sex and Gender in Research

Cell line source(s)	Hi5 cells: Expression Systems (#94-002F), Tni insect cells in ESF921 media.<br>Saccharomyces cerevisiae: RSC2-TAP-HIS3 (YSC1177-YLR357W), Dharmacon, TAP-tagged open reading frame library.<br>Saccharomyces cerevisiae: INO80 overexpression strain (yAE86), obtained from Kurat et al. Molecular Cell 2017).
Authentication	Authentication of the yeast strains was performed by PCR. The insect cells were authenticated by the manufacturer.
Mycoplasma contamination	The insect and yeast cell lines used for protein expression were not tested for mycoplasma contamination.
Commonly misidentified lines<br>(See ICLAC register)	None.

ChIP-seq

Data deposition

☒ Confirm that both raw and final processed data have been deposited in a public database such as GEO.

☒ Confirm that you have deposited or provided access to graph files (e.g. BED files) for the called peaks.

Data access links<br>*May remain private before publication.*	http://www.ncbi.nlm.nih.gov/geo/ via GEO accession number GSE220647 (token: wnyncsemtjybfsp)
Files in database submission	Fastq files and bigwig files for the following samples were uploaded to GEO:<br><br>Replicate1_ control_IP_Reb1<br>Replicate1_TFs-only_IP_Reb1<br>Replicate1_INO80_IP_Reb1<br>Replicate1_ISW2_IP_Reb1<br>Replicate1_Chd1_IP_Reb1<br>Replicate1_RSC_IP_Reb1<br>Replicate2_ control_IP_Reb1<br>Replicate2_TFs-only_IP_Reb1<br>Replicate2_INO80_IP_Reb1<br>Replicate2_ISW2_IP_Reb1<br>Replicate2_Chd1_IP_Reb1<br>Replicate2_RSC_IP_Reb1<br>Replicate1_ control_IP_Abf1<br>Replicate1_TFs-only_IP_Abf1<br>Replicate1_INO80_IP_Abf1<br>Replicate1_ISW2_IP_Abf1<br>Replicate1_Chd1_IP_Abf1<br>Replicate1_RSC_IP_Abf1<br>Replicate2_ control_IP_Abf1<br>Replicate2_TFs-only_IP_Abf1<br>Replicate2_INO80_IP_Abf1<br>Replicate2_ISW2_IP_Abf1<br>Replicate2_Chd1_IP_Abf1<br>Replicate2_RSC_IP_Abf1<br>Replicate1_ control_Input_Reb1<br>Replicate1_TFs-only_Input_Reb1<br>Replicate1_INO80_Input_Reb1<br>Replicate1_ISW2_Input_Reb1<br>Replicate1_Chd1_Input_Reb1<br>Replicate1_RSC_Input_Reb1<br>Replicate2_ control_Input_Reb1<br>Replicate2_TFs-only_Input_Reb1<br>Replicate2_INO80_Input_Reb1<br>Replicate2_ISW2_Input_Reb1<br>Replicate2_Chd1_Input_Reb1<br>Replicate2_RSC_Input_Reb1<br>Replicate1_ control_Input_Abf1<br>Replicate1_TFs-only_Input_Abf1

Replicate1_INO80_Input_Abf1
Replicate1_ISW2_Input_Abf1
Replicate1_Chd1_Input_Abf1
Replicate1_RSC_Input_Abf1
Replicate2_ control_Input_Abf1
Replicate2_TFs-only_Input_Abf1
Replicate2_INO80_Input_Abf1
Replicate2_ISW2_Input_Abf1
Replicate2_Chd1_Input_Abf1
Replicate2_RSC_Input_Abf1

Genome browser session (e.g. UCSC)

https://tinyurl.com/yurw2hwf

Methodology

Replicates	Two biological replicates for each experimental condition were performed.
Sequencing depth	The samples were sequenced using the Illumina NextSeq550 sequencer in 42 bp paired-end mode to a sequencing depth of ~5 Mio. reads per sample.
Antibodies	Strep-Tactin® (IBA, 2-1613-002). Please note that Strep-Tactin® (modified Streptavidin) was used instead of an Antibody.
Peak calling parameters	MACS2 (default parameters).
Data quality	The quality of the in vitro Reb1 and Abf1 ChIP-seq data was assessed by comparing peaks and discovered PWM motifs to previously published in vivo Reb1 and Abf1 ChIP-seq data sets and motifs (Gutin et al. 2018, Cell Reports; Rossi et al. 2021, Nature).
Software	Reads were mapped to the SacCer3 genome (R64-1-1 assembly) using Bowtie, omitting multiple matches. Peak calling was performed with MACS2 and motif discovery in the regions of the called peaks was performed using MEME.
