# Supplementary figures and images for: In vitro reconstitution of chromatin domains shows a role for nucleosome positioning in 3D genome organization
Source: Nat Genet. 2024 Jan 30;56(3):483–92. doi: 10.1038/s41588-023-01649-8 (PMC10937381; doi:10.1038/s41588-023-01649-8)

histone octamers

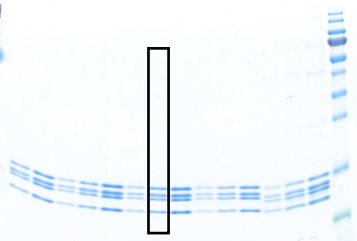

Abf1 Reb1

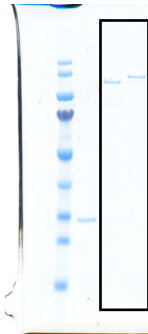

Chd1

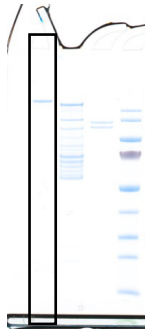

ISW2

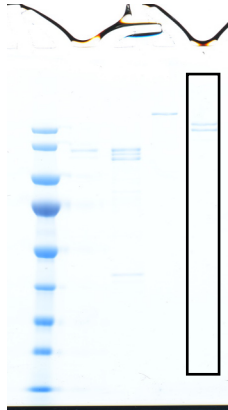

INO80

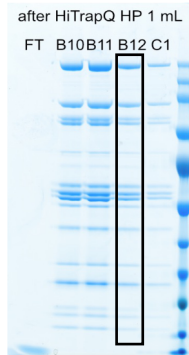

RSC

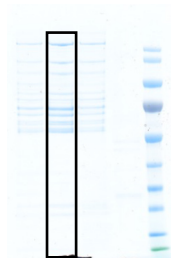

Supplement: Supplementary file 7 — Uncropped and raw SDS–PAGE gels. [file 41588_2023_1649_MOESM7_ESM.pdf]

Control Chd1 ISW2 INO80

D D D D

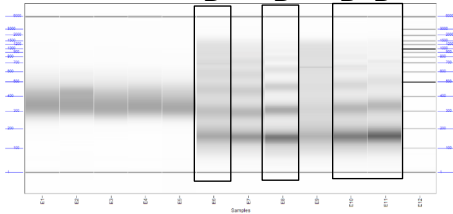

Control Chd1 ISW2 INO80

L L L L

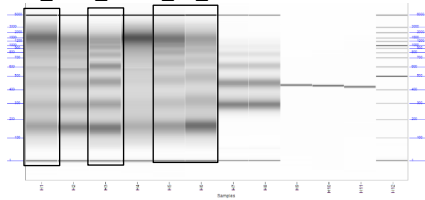

RSC

D

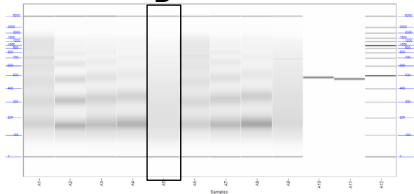

RSC

L

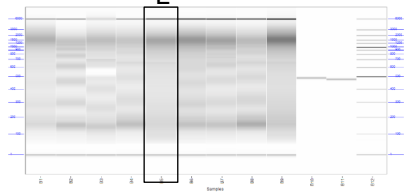

Supplement: Supplementary file 8 — Uncropped Fragment Analyzer images. [file 41588_2023_1649_MOESM8_ESM.pdf]

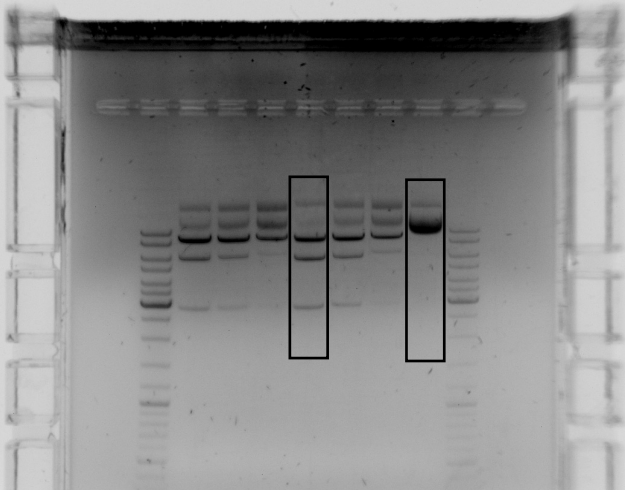

Exp. Time: 0.88 sec Upper: 100 % Lower: 0 %

Date: 28.03.2023 Time: 09:20:30

Supplement: Supplementary file 9 — Uncropped agarose gels. [file 41588_2023_1649_MOESM9_ESM.pdf]
